# Spent coffee grounds as feedstock for the production of biosurfactants and the improved recovery of melanoidins

**DOI:** 10.1007/s11274-023-03698-x

**Published:** 2023-07-18

**Authors:** Ignacio Moya-Ramírez, María Encarnación Pegalajar-Robles, Michele Debiasi Alberton, José A. Rufián-Henares, Alejandro Fernández-Arteaga, Miguel Garcia-Roman, Deisi Altmajer-Vaz

**Affiliations:** 1 Departamento de Ingeniería Química, Universidad de Granada, Avda. Fuentenueva s.n, Granada, 18071 Spain; 2 Departamento de Ciências Farmacêuticas, Universidade Regional de Blumenau, Blumenau, Brasil; 3 Departamento de Nutrición y Bromatología, Instituto de Nutrición y Tecnología de los Alimentos, Centro de Investigación Biomédica and Instituto de Investigación Biosanitaria ibs.GRANADA, Universidad de Granada, Granada, 18100 Spain

**Keywords:** Antioxidant capacity, Biosurfactant, Melanoidins, Phenols, Spent coffee grounds, Surfactin

## Abstract

Spent coffee grounds (SCG) are wastes generated in high amounts worldwide. Their composition makes them a promising feedstock for biotechnological processes. Here we show that the production of the biosurfactant surfactin by submerged culture of a *Bacillus subtilis* strain growing on SCG is possible, reaching concentrations up to 8.8 mg/L when using SCG at 8.3 g/L in the medium. In addition, we report a synergy between the production of surfactin and the recovery of melanoidins, an added-value compound already present in SCG. More specifically, the concentration of melanoidins in the culture medium increased between 2.1 and 2.5 times thanks to the presence of the *B. subtilis* in the culture. Furthermore, we have observed a strong interaction between surfactin and melanoidin aggregates through dynamic light scattering measurements, and that both of them can be co-purified with an acid precipitation. We have also characterized the interfacial and antioxidant properties of the cell-free supernatant and surfactin extract, as well as the distribution of the congeners of the biosurfactant. Altogether, this work describes a promising approach to obtain biosurfactants and antioxidant molecules in a single operation, which can be used to design several new formulations of interest for bioremediation, amendment of soils, food and cosmetics.

**Supplementary Information:**

The online version contains supplementary material available at 10.1007/s11274-023-03698-x.

## Introduction

The progress of our society towards a circular economy is attracting an increasing social and scientific consensus. This is pushing manufacturers to use raw materials obtained from renewable sources and extend the permanence of these materials in the production cycle by reusing and recycling them. In this context, biotechnology plays a crucial role in the advance to a more sustainable industrial system, since it enables to employ a wide variety of products, useless for traditional industries, as feedstocks for the production value-added biomolecules. Probably, the most relevant raw material for bioprocesses are plant-derived agro-industrial and food wastes. These are rich in organic molecules such as carbohydrates, mono- and oligosaccharides, aromatic compounds or oils, which can be valorized, for example, as carbon sources for the culture of microorganism and the production of biomolecules such as key chemicals or enzymes (Panda et al. 2016; Lee et al. 2019).

Spent coffee grounds (SCG) are one of these prominent sources of agro-industrial wastes. These are generated as leftovers after the extraction of the raw coffee powder with hot water or steam. Around 10 million tons of coffee are produced annually according to the international coffee organization, and it is estimated that an average of 650 kg of SCG are produced per ton of green coffee beans (Murthy and Madhava Naidu 2012). Currently SCG have a low value and are normally disposed in landfill or incinerated (Karmee 2018). However, given their composition and availability, they represent a potentially relevant raw material for biomanufacturing. SCG are a rich source of carbohydrates, in particular mannose, galactose and glucose, the main components of its hemicellulose fraction (Karmee 2018). They also are rich in oil, proteins and bioactive compounds of interest such as polyphenols and melanoidins (Araújo et al. 2019; Battista et al. 2020; Leal Vieira Cubas et al. 2020; Ramírez et al. 2021; Ribeiro et al. 2021). Significantly, melanoidins from SCG have gained a considerable attention due to their antioxidant, antimicrobial and anticancer activity, as well as their capacity to chelate metal ions (Langner and Rzeski 2014). Furthermore, several recent studies have used SCG as raw material for the production of biochemicals, focusing on the production of enzymes, polyhydroxyalcanoates, polyphenols, biofuels or bio-composites (Cervera-Mata et al. 2020; Battista et al. 2020; Mendoza Martinez et al. 2021; Lee et al. 2021). In addition to these, biosurfactants are a family of biomolecules that have an increasing relevance in biomanufacturing. Biosurfactants are surface active molecules produced by microorganisms that stand as a real alternative to petroleum-derived synthetic surfactants since they have better biocompatibility and wider structural diversity and applicability. More importantly, biosurfactants are compatible with sustainable production chains (Singh et al. 2019). Despite that, the production of biosurfactants from SCG has been barely explored, with a single study reported to date by Yañez-Ocampo et al. in 2017 (Yañez-Ocampo et al. 2017).

Therefore, in this work we propose the production of biosurfactants as a valorization route for SCG. For that, we aim to analyze the feasibility of using SCG as the carbon source for the culture of the bacterium *Bacillus subtilis* DSM 3526, a strain producer of the biosurfactant surfactin. We have studied the time course of the *B. subtilis* cultures fed with different concentrations of SCG and characterized in detail the yield of surfactin and the distribution of its congeners by UPLC-MS. Remarkably, in addition to the production of biosurfactants, we report for the first time how the production of surfactin leads to an increase of the amount of melanoidins that can be recovered from the spent coffee grounds. We have also measured the interfacial and antioxidant properties of the cell-free supernatant and the melanoidins extracted from these cultures. Finally, we have analyzed the interaction between surfactin and melanoidins by dynamic light scattering, in order to understand the interaction between biomolecules, which are simultaneously produced and recovered in a single operation.

## Experimental section

### Materials

Culture media components and other reagents used were purchased form Merk (Darmstadt, Germany). Two surfactin standards were used in this work, one HPLC-grade surfactin (Merk, purity ≥ 98%) as reference for the UPLC-MS analyses, and a sodium surfactin purchased from Kaneka (Osaka, Japan, purity > 90%) used for the DLS and CMC experiments. Spent coffee grounds (SCG) were kindly supplied by a local coffee shop (Granada, Spain). Before their use, SCG were dried at 100ºC for 24 h, reducing its moisture content to less than 2%. The SCG where characterized as follows: (i) the total lipids were measured by a Soxhlet extraction with petroleum ether at 40–60 ºC after a hydrolysis with HCl 4 N. The free fatty acids profile was determined by a methylation of the extract followed by gas chromatography Agilent 7890 A chromatograph (Agilent, Ca., USA); (ii) the protein content was measured by a Kjeldahl digestion, using a 6.25 factor to convert the total Kjeldahl nitrogen to protein; (iii) an elemental analysis was performed with a Thermo Scientific™ FLASH 2000 CHNS/O Analyzer (Thermo Fisher Scientific, Ma., USA).

### Bacterium strain and biosurfactant production and characterization

*Bacillus subtilis* DSM 3526 was acquired from the German Collection of Microorganisms and Cell Cultures. The strain was stored at -80ºC in a nutrient medium comprised of 2.0 g/L of yeast extract, 1.0 g/L of beef extract, 5.0 g/L of peptone, 5.0 g/L of sodium chloride and 20% (v/v) of glycerol. The frozen stocks of *B. subtilis* DSM 3526 were streaked in a nutrient agar Petri dish and incubated for 24 h at 30 ºC. Next, one colony was used to inoculate a 250 mL Erlenmeyer flask containing 50 mL of medium comprised of 2.0 g/L of yeast extract, 1.0 g/L of beef extract, 5.0 g/L of peptone and 5.0 g/L of sodium chloride. This inoculum culture was incubated at 30ºC and 160 rpm for 24 h. Experiments with *B. subtilis* DSM 3526 growing in SCG were performed in 500 mL Erlenmeyer flasks containing 190 mL of a culture medium based on the described by Vedaraman and Venkatesh (Vedaraman and Venkatesh 2011): 5.0 g/L of yeast extract, 1.0 g/L of KH_2_PO_4_, 0.5 g/L of MgSO_4_·7H_2_O, 0.1 g/L of CaCl_2_, 0.1 g/L of NaCl, 0.7 g/L of peptone, and SCG ranging from 8.3 g/L to 24.9 g/L in dry base. The pH of the medium was adjusted to 7.2 with NaOH 1 M and autoclaved at 121 ºC for 15 min. The flasks were inoculated with 10 mL of the inoculum culture adjusted to an OD_600_ of 0.8 with fresh medium and incubated at 30ºC on a rotary shaker at 160 rpm. Control cultures were performed without the addition of SCG or *B. subtilis* inoculum. At the selected timepoints cultures were sampled and cells separated by centrifugation at 10^4^xg for 10 min at 4 ºC. The cell-free supernatant samples were processed and analyzed as described below.

#### Surfactin recovery, purification and characterization

Surfactin was recovered from the cell-free supernatant (CFS) by acid precipitation. For that, the pH of the CFS was adjusted to 2.0 with 6 M HCl and kept at 4 ºC overnight. The precipitated biosurfactant was separated by centrifugation at 10^4^ x g for 20 min at 4 ºC, resuspended in a small amount of distilled water and adjusted to pH 7.0 with 1 N NaOH. Finally, the recovered surfactin extract was lyophilized, weighed, and stored at -18ºC. The powder obtained in this way is referred as crude surfactin (CrS). CrS production experiments were performed at least as biological duplicates.

The concentration and congener distribution of surfactin on the CFS and CrS was measured by ultra-high pressure liquid chromatography coupled to mass spectrometry (UPLC-MS) in a UPLC Waters Acquity H-Class chromatograph (Waters Corporation, Milford-MA, USA) equipped with a Waters UPLC BEH C-18 column and coupled to a mass spectrometer (Waters Xevo-TG-S). The mobile phase was composed by a mixture of 20% of solvent A (water with 0.1% w/w of formic acid) and 80% of solvent B (acetonitrile with 0.1% w/w of formic acid). Surfactin congeners with a variable number of carbons on their β-hydroxy fatty acid chain were identified (C12 to C16), as shown in figure S1 (supporting information), whose identification was validated with the HPLC-grade surfactin standard (purity ≥ 98%) as shown in figure S1-e. This standard was used also to build a calibration curve (Figure S2) in order to quantify the concentration of surfactin in the supernatants. CFS samples were directly injected into the chromatograph, and CrS samples were previously dissolved in a known volume of methanol.

#### Interfacial properties of the crude surfactin

Surface tension (ST) of the CFS was measured at 25 ºC with a Wilhelmy plate in a KRUSS K11 Tensiometer (KRÜSS GmbH, Hamburg, Germany). For that, CrS solutions at different concentrations were prepared in milliQ-grade water adjusting their pH to 7.0 with 1 M NaOH. The critical micelle concentration (CMC) of CrS was determined at 25ºC, based on the break point of the ST vs. CrS concentration plot. Interfacial tension (IT) between aqueous solutions of CrS and engine oil (Repsol, Spain) and contact angle (CA) between drops of CrS solutions and Parafilm®-coated glass sheets were determined at 25 ºC in a pendant drop tensiometer (KSV CAM 200, KSV Instruments Ltd., Finland). In addition, the emulsification index (EI) was studied following the protocol described by Cooper & Goldenberg (Cooper and Goldenberg 1987). For that, 2 mL of a CrS solution (at concentrations ranging its CMC value) and 2 mL of hexadecane were vortexed in test tubes for 2 min at the highest speed. The resulting emulsions were incubated at 25 ºC. To calculate de EI the height of emulsion after 1 and 8 days was divided by the height at t = 0. All the experiments were carried out by triplicate.

### Analytical procedures

The following measurements were carried out in the SCG and the cell–free supernatant (CFS):

The concentration of soluble carbohydrates in the CFS was measured with the phenol-sulfuric acid method, as described by Nielsen (Nielsen 2010). The content of melanoidins in the CFS was determined through their absorbance at 420 nm (Yen et al. 2005). A calibration curve was prepared with extracted melanoidins (Figure S3) as previously described by Rufián-Henares and de la Cueva (Rufián-Henares and de La Cueva 2009), which have a molecular weight ranging between 21 and 28 kDa according to high-performance gel permeation chromatography measurements (Figure S4). The interaction between surfactin micelles and melanoidin aggregates was studied by dynamic light scattering in a Zetasizer Ultra (Malvern Panalytical, UK) at 25 ºC. For that, 1 g/L solutions of sodium surfactin and melanoidins extracted from SCG were prepared in 0.1 M phosphate buffer at pH 7.4, and filtered with 0.22 μm syringe filters. The concentration of the total of phenolic compounds (PC) in the CFS and CrS was analyzed by the Folin-Ciocalteu method (Anagnostopoulou et al. 2006). The PC content was expressed in terms of mg of gallic acid/mL of CFS or as mg gallic acid/gram of CrS.

Finally, the antioxidant capacities of CFS and CrS were evaluated by four different methods: (i) the DPPH (2,2-diphenyl-1-picrylhydrazyl) free radical scavenging activity, following the procedure described by Pastoriza et al.(Pastoriza and Rufián-Henares 2014) (ii) the inhibitor potential of the lipid peroxidation (ILP) using as model β-carotene/linoleic acid, according to Mokbel and Hashinaga (Mokbel and Hashinaga 2006); (iii) the iron reducing power described by Moreno-Montoro et al.(Moreno-Montoro et al. 2015) and, (iv) the chelating activity on ferrous ions (Fe^2+^), as described by Kilic (Kilic et al. 2014). As positive controls, gallic acid 1000 µg/mL was used for the DPPH method; 1000 µg/mL butylated hydroxytoluene (BHT) for the ILP method, and 100 µg/mL EDTA for Fe^2+^ chelating assay. The iron reduction potential was expressed as mg of gallic acid/g of CrS (mg/g), or mg of gallic acid/mL of CFS (mg/mL). All the measurements were performed in triplicate.

## Results

### Spent coffee grounds as carbon source for microbial culture

We characterized the spent coffee grounds (SCG) used in this work by an elemental analysis together with analyses of its lipids, free-fatty acids, proteins, ash, total phenols and melanoidins content, as shown in Table 1. All of them were in good agreement with previous works on SCG (Kondamudi et al. 2008; Mussatto et al. 2011a, b; Pujol et al. 2013; Abdullah and Bulent Koc 2013). These data show that SCG is a complex mix of different substrates, with a high content of molecules susceptible to be metabolized by microorganism such as polysaccharides, which represent around a 12.4% for cellulose and 39.1% for hemicellulose (Ballesteros et al. 2014) or proteins and lipids, each of them representing aound a 15% in dry base a shown in Table 1.

**Table 1:** Chemical composition of the spent coffee grounds used as carbon source for the culture of microorganism and biosurfactant production

Parameter		Value
Elemental analysis		C (%): 51.44
		H (%): 7.75
		N (%): 2.29
Lipid content		15.0 ± 0.2% (db)
Protein content		14.7 ± 1.0% (db)
Total phenolic compounds		0.18 ± 0.02 mg/g**
Ash content (combustion at 550ºC)		1.08% (db)
Free fatty acids (%) CG-FID	Palmitic acid (C16:00)	32.1 ± 6.2
	Stearic acid (C18:00)	6.3 ± 0.0
	Oleic acid (C18:1n-9c)	9.6 ± 0.8
	Linoleic acid (C18:2n-6c)	31.7 ± 8.7
	γ-Linolenic acid (C18:3n-6c)	1.9 ± 0.5
	Linolenic acid (C18:3n-3c)	0.8 ± 1.1
	Henecosanoic acid (C21:00)	0.7 ± 0.4
	Behenic acid C22:00)	3.5 ± 3.9
	Erucic acid (C22:1n-9)	1.9 ± 2.0
	Tricosanoic acid (C23:00)	3.4 ± 3.2
	Lignoceric acid (C24:00)	1.2 ± 1.5
	Nervonic acid (C24:1n-9)	7.0 ± 6.4

*db: dry basis. ** Results of total phenols are in mg gallic acid/g of SCG.

Considering that, we tested whether SCG can be upgraded by their use as a substrate for the culture of microorganisms and the production of valuable biomolecules. For that, we grew *B. subtilis* DSM 3526 in submerged cultures at various concentration of SCG. *B. subtilis* is a well-known producer of the biosurfactant surfactin, a lipopeptide composed by a hydrophilic ring of 7 amino acids and a hydrophobic β-hydroxy fatty acid chain of variable length.

**Fig. 1:**
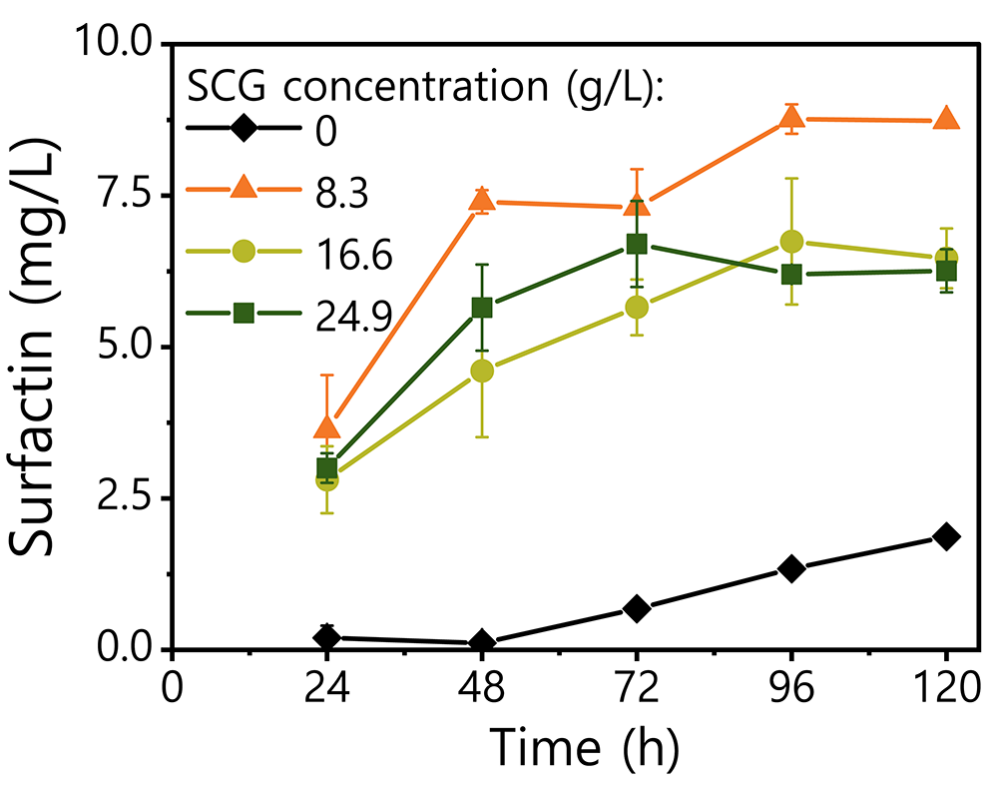
Surfactin concentration in the cell free supernatant (CFS) of *B. subtilis* DSM 3526 cultures with different concentrations of spent coffee grounds (SCG) in the medium. Cultures were sampled at each time-point, the cells removed and the surfactin concentration in the CFS measured by UPLC-MS. The results show the average and standard deviation of at least a biological duplicate

We tested three SCG concentrations, and for all of them the *B. subtilis* produced approximately 3 mg/L of surfactin during the first 24 h, as shown in Fig. 1. The main production of surfactin took place during the first 48 h of culture and reached the maximum concentration after 96 h for all the three SCG concentration tested. Considering the effect of the amount of SCG in the medium, cultures with 8.3 g/L of SCG yielded the highest surfactin concentration, 8.8 mg/L after 96 h, almost a 30% higher than the cultures with SCG at 16.6 g/L and 24.9 g/L, while no significant differences in the yield of surfactin were observed between these two highest SCG concentrations. Therefore, these results suggest that there is no improvement in the titer of surfactin when the medium is supplemented with SCG above 8.3 g/L. On the contrary, it seems that the production of surfactin is hampered at higher SCG concentrations, which suggest some inhibitory effect, which could be attributed to the phenolic substances present in SCG.

As shown in Fig. 1, surfactin is a secondary metabolite produced during the late-exponential and early-stationary growth phases. Similar trends have been previously reported for *B. subtilis* strains grown with glucose or other agricultural wastes as carbon sources (Sen 1997; Maass et al. 2016). Indeed, the total soluble carbohydrates concentration in the CFS at the beginning of the culture was 1.41 g/L, and their metabolization took place during the first 24 h of culture, decreasing to 0.73 g/L, which remained unaltered afterwards (Table 2). The carbohydrates present in in control cultures where no SCG were added, probably present in the yeast extract used in the medium, showed the same consumption trend. Interestingly, despite the control medium supported the growth of the *B. subtilis*, the dynamic in the production of surfactin was different and the yield considerably lower compared to the media with SCG. In this case, 1.3 mg/L of surfactin were produced after 96 h, 6.8 times lower than with 8.3 g/L of SCG in the culture medium. This confirms that the bioavailable molecules in the SCG are metabolized by the bacteria and contribute to the biosynthesis of surfactin.

**Table 2:** Total soluble carbohydrates content in the cell free supernatant of *B. subtilis* cultures at different times and SCG concentrations

Culture medium	Time (h)	Soluble carbohydrates content (g/L)
SCG = 8.3 g/L	0	1.41 ± 0.03
SCG = 8.3 g/L	24	0.73 ± 0.03
SCG = 8.3 g/L	48	0.73 ± 0.03
SCG = 0 g/L	0	0.54 ± 0.01
SCG = 0 g/L	48	0.27 ± 0.01

#### Distribution of the surfactin congeners

**Fig. 2:**
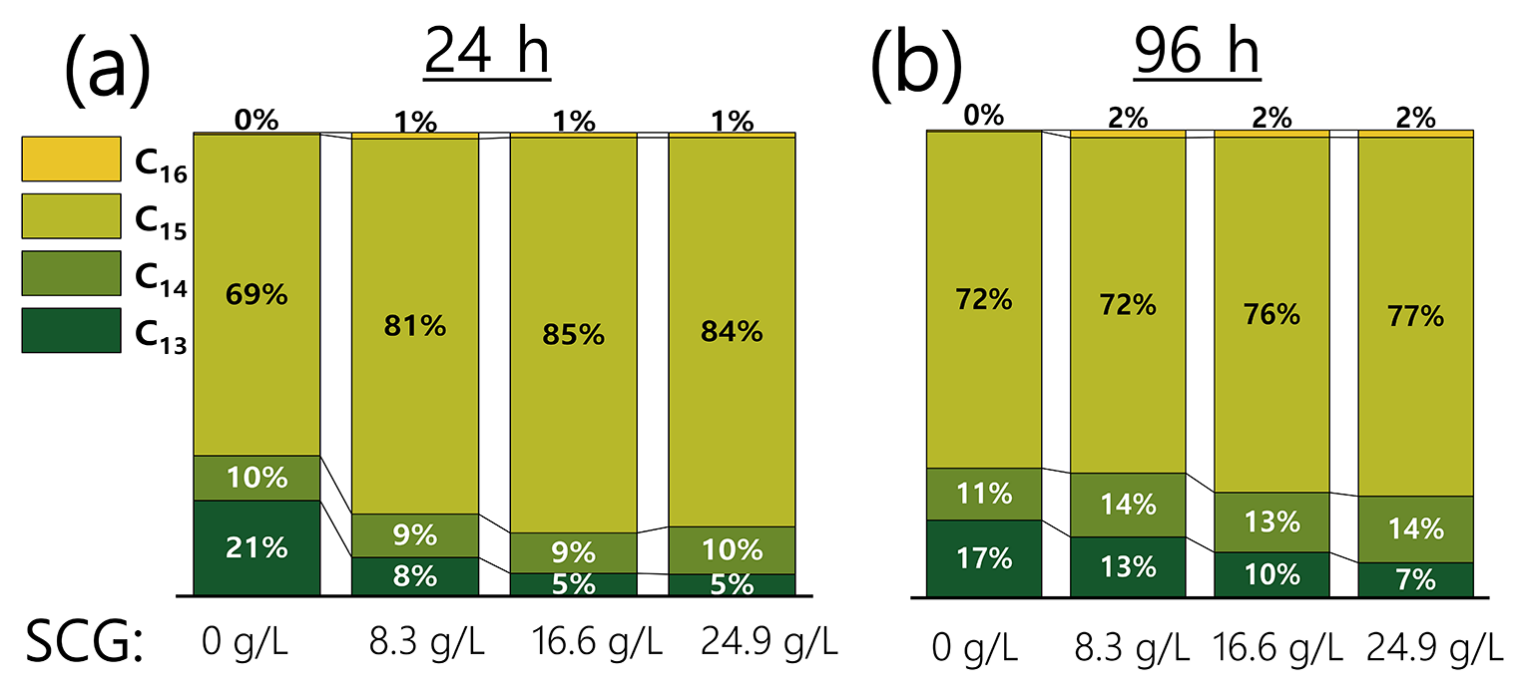
Percentages of each of the surfactin congeners in the CFS after 24 and 96 h of culture at different concentrations of SCG in the culture media measured by UPLC-MS. The length of the β-hydroxy fatty acid chain of the congeners detected varied from 13 to 16 carbon atoms (congeners named as C_13_ to C_16_). Standard deviations correspond to at least a biological duplicate of the experiment

Four different surfactin congeners were detected by UPLC-MS, differing from each other in the number of carbon atoms of their β-hydroxy fatty acid chain (Fig. 2 and S1). For all the condition tested, C_15_ surfactin was the more abundant congener, representing between a 69% and 85% of the total biosurfactant produced, followed by the C_14_ and C_13_ species and a marginal amount of C_16_. This profile remained mostly unaltered between the 24 and 96 h of culture, even so a slight decrease on C_15_ and an increase on C_14_ and C_13_ was observed for all the cultures with SCG in the medium. On the other hand, for the control experiment (without SCG) the congeners distribution was different, showing higher amounts of C_13_ surfactin.

Considering these observations and previous work of our group with the same *B. subtilis* strain, we conclude that there is a direct influence of the carbon source over the distribution of surfactin congeners. In particular, we observed that the use of SCG increased the fraction of C_15_ surfactin and reduced the C_16_ one, compared to when waste frying oil is used as carbon source (Valenzuela Ávila et al. 2019). Similarly, the high lipid content of the SCG compared to the control cultures with no SCG could be responsible of the shift observed here, given that the fatty acid biosynthesis pathway is involved on the supply of surfactin precursors (Zhi et al. 2017; Wu et al. 2019). The distribution of the surfactin congeners is relevant, since the length of the hydrophobic chain affects the interfacial behavior of a surfactant (Liu et al. 2012, 2015). Thus, these results show that SCG can be used to tune the properties of the surfactin produced, as previously reported for other carbon sources (Raza et al. 2009; Soares da Silva et al. 2017).

### Properties of the culture medium and the surfactin extract obtained from SCG

Crude surfactin (CrS) was extracted from the CFS by acid precipitation. The concentration CrS was in the range of 1.55 g/L and 3.35 g/L after 48 h of culture with 8.3 g/L and 16.6 g/L of SCG respectively, considerably higher than the surfactin concentration measured by UPLC-MS. This means that the purity of surfactin extracted with acid precipitation is low, representing a 0.48% and 0.14% of the crude extract respectively. Considering that the yields of surfactin were similar for both concentrations of SCG, the sources of the impurities co-purified with the surfactin would be components of SCG that remain in the medium during the microbial culture. Therefore, further purification steps would be required if SCG are to be used as carbon source for the production of pure surfactin.

However, these co-purified substances might have an additional added- value to the surfactin produced from SCG. In particular, the melanoidins and phenolic substances are susceptible to form part of these impurities in the CrS, and can confer CrS antioxidant properties. Therefore, it is relevant to study the interaction of melanoidins with surfactin and the antioxidant capacity of CrS obtained from SCG, particularly considering that these have been barely analyzed for surfactin produced from plant-derived agro-industrial and food wastes so far.

#### Interfacial properties of CrS

**Fig. 3:**
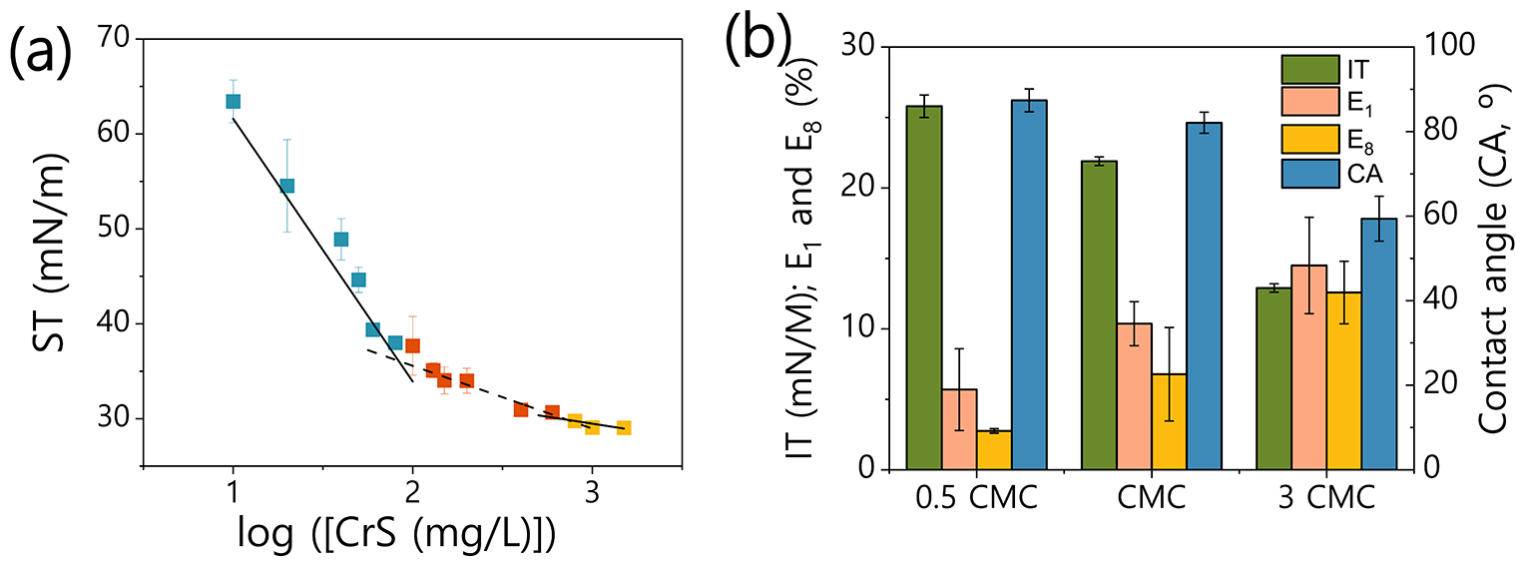
Interfacial properties of the crude surfactin (CrS) recovered after 48 h from cultures with 8.3 g/L of SCG. **(a)** The surface tension of succesive dilutions of CrS (ploted as the log_10_ of its concentraion in mg/L) was used to determine its critical micelle concentrations (CMC) by the intersection of the linear fiting of the points belonging to three regions of the plot; **(b)** Contact angle (CA), interfacial tension (IT) and hexadecane emulsion index after 1 and 8 days after the preparation (EI_1_ and EI_8_). Average values and standard deviation of experimental triplicates are shown

In order to describe the interfacial properties of the CrS, we have initially determined its critical micelle concentration (CMC). According to the ST vs. CrS concentration plots (Fig. 3.a), the CMC of the CrS is 83.4 mg/L, which corresponds to a ST of 36.1 mN/m. This value is in the same order of magnitude of other CMCs found for surfactin extracts previously, although it is higher compared to most of them (Zeraik and Nitschke 2010; Vaz et al. 2012). It is also higher than the CMC values reported for pure surfactin, which is around 10 mg/L (Ishigami et al. 1995; Qin et al. 2023). It is important to consider that the CMC of the CrS is highly influenced by its purity and the nature of the impurities on it. Thus, since the fraction of surfactin in the CrS produced is low, high CMC values are expected. Even more, a second CMC seems to occur around a CrS concentration of 723.4 mg/L and a ST of 29.9 mN/m. This support the hypothesis that impurities with interfacial properties (such as protein/peptides or phenolic compounds) are present in the CrS and can interact with the surfactin and its interfacial arrangement.

Considering that, we tested whether melanoidins could interact with surfactin and modify the CMC of the biosurfactant. For that, we measured the CMC of a commercial surfactin (Kaneka, purity > 90%) in the presence of a fixed concentration of melanoidins at 2.75 g/L and in the absence of it. As can be observed in Figure S5, the presence of melanoidins in the solution did not modify the CMC of the surfactin, which was around 10 mg/L for and 28 mN/m both cases. Thus, the presence of melanoidins in the CrS seem not to be responsible its high CMC, and neither originate the possible second CMC found. Further experimentation would be necessary to explore the effect of other impurities on the CrS, such as extracellular proteins or peptides, which could modify the interfacial behavior of the biosurfactant.

Next, we evaluated the contact angle (CA), interfacial tension (IT) and emulsification index (EI) of dilutions of CrS at concentrations around its CMC. As expected, all of these parameters describing the interfacial properties of CrS were strongly affected by its concentration (Fig. 3.b). The contact angle of the solutions of CrS varied from 87.4 ± 2.7° at 0.5 times of its CMC to 59.4 ± 5.3° at 3 times its CMC. This decrease in the contact angle indicates the excellent ability of the CrS to wet a hydrophobic surface. The wettability of these solutions was higher than for example those found for surfactin produced from olive oil mill wastes, which had a CA of 76.74° at 3xCMC (Maass et al. 2016). The interfacial tension between CrS solutions and engine oil ranged between 12 and 24 mN/m in the range of concentrations tested, values similar to those found for other crude surfactin extracts (Deleu et al. 1999; Al-Wahaibi et al. 2014; de Oliveira et al. 2016; Maass et al. 2016). Lastly, CrS showed a limited emulsifying power at the concentration tested, reaching values of emulsion index after 1 day (EI_1_) of 14.5 at 3xCMC. Again, this could be explained considering the low percentage of active emulsifier, i.e., surfactin, in the CrS. However, despite these low EI_1_ values, most of the emulsified hexadecane remained in this condition eight days after its preparation, in particular for the x3 CMC concentration. Therefore, it could be possible to obtain stable emulsions during extended periods of time using the CrS produced by the method described here, which would need however an optimization to increase their EI by, for example, increasing the concentration of CrS or adding adjuvants.

#### Melanoidins content in the CFS and their interaction with surfactin

Melanoidins are anionic macromolecules formed by the reaction of reducing sugars and amino acids during the Maillard reaction (Rufián-Henares et al. 2006; Delgado-Andrade et al. 2007), and thus present in thermally processed foods such as roasted coffee (Rufián-Henares and de La Cueva 2009; Pastoriza and Rufián-Henares 2014), giving it its characteristic intense brown color (Rufian-Henares et al. 2002). They are of interest as food supplement given their antimicrobial, anticancer and antioxidant properties (Langner and Rzeski 2014). This last feature is of particular relevance, and it is attributed to two different mechanisms: (i) the metal chelating ability (Ćosović et al. 2010; Wang et al. 2011a), and (ii) the radical-scavenging activity (Sacchetti et al. 2009; Pastoriza and Rufián-Henares 2014).

**Fig. 4:**
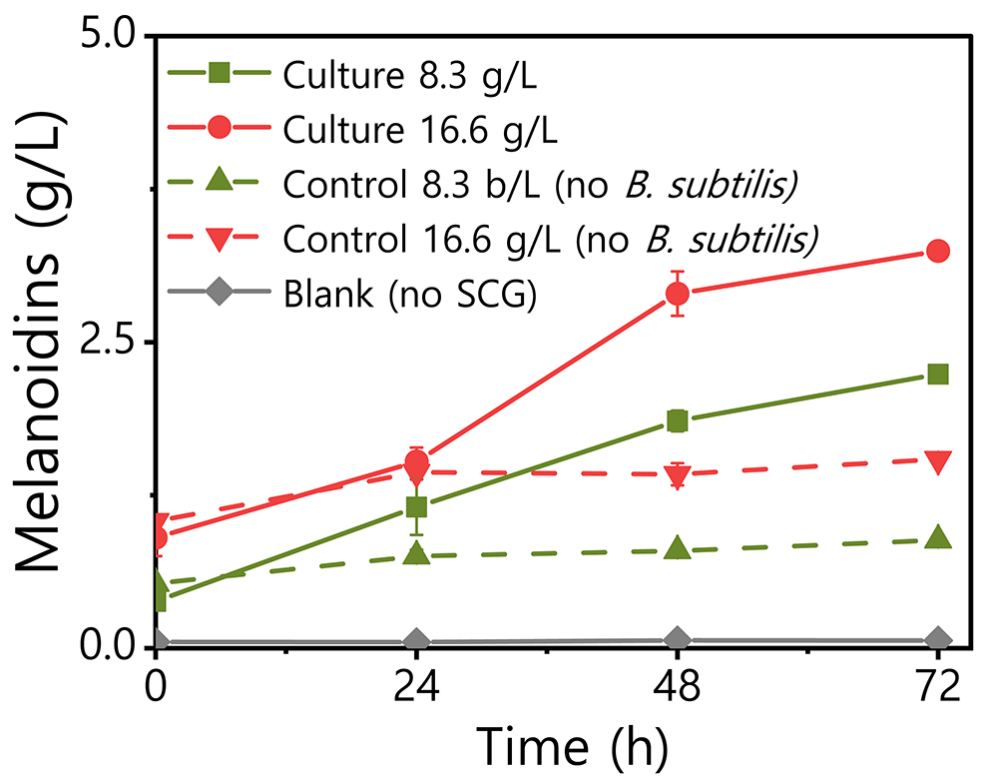
Evolution of the concentration of melanoidins in the cell-free supernatant of *B. subtilis* cultures growing in media with SCG at 8.3 and 16.6 g/L. Control experiments with the same SCG concentrations but where the *B. subtilis* was not inoculated where also performed, together with a blank culture where no SCG was added to the medium. Average values and standard deviation of experimental triplicates are shown

We have therefore analyzed in depth the concentration of melanoidins and the antioxidant properties of the cell-free supernatant and crude surfactin extract. We found that the concentration of melanoidins in the CFS of *B. subtilis* cultures growing on SCG increased during the first 48–72 h of culture (Fig. 4). Interestingly, the concentration of melanoidins in the CFS from bacterial cultures was notably higher compared to negative control experiments where the bacterium was not inoculated. These controls had a constant melanoidin concentration during the period of time analyzed. More specifically, the concentration of melanoidins in CFS increased 2.5 and 2.1 times compared to their negative controls where the bacterium was not inoculated, for the experiments carried with 8.3 g/L and 16.6 g/L of SCG respectively. In addition to that, the concentration of melanoidins was proportional to the amount of SCG in the media for both, cultures and controls. Hence, these results show for first time that the extraction of melanoidins from the SCG can be improved simply by a bacterial culture. This effect could be promoted by the action of surfactin as interfacial agent, which would contribute to their extraction from the solid SCG to the liquid medium. Besides, we estimate that the acid precipitation used to recover the surfactin from the CFS extracted the 66.7% and 74.0% of the melanoidins initially present on the CFS after 24 and 48 h of culture respectively. Therefore, it is possible to produce and recover both added-value bioproducts with a single microbial culture and purification step.

**Fig. 5:**
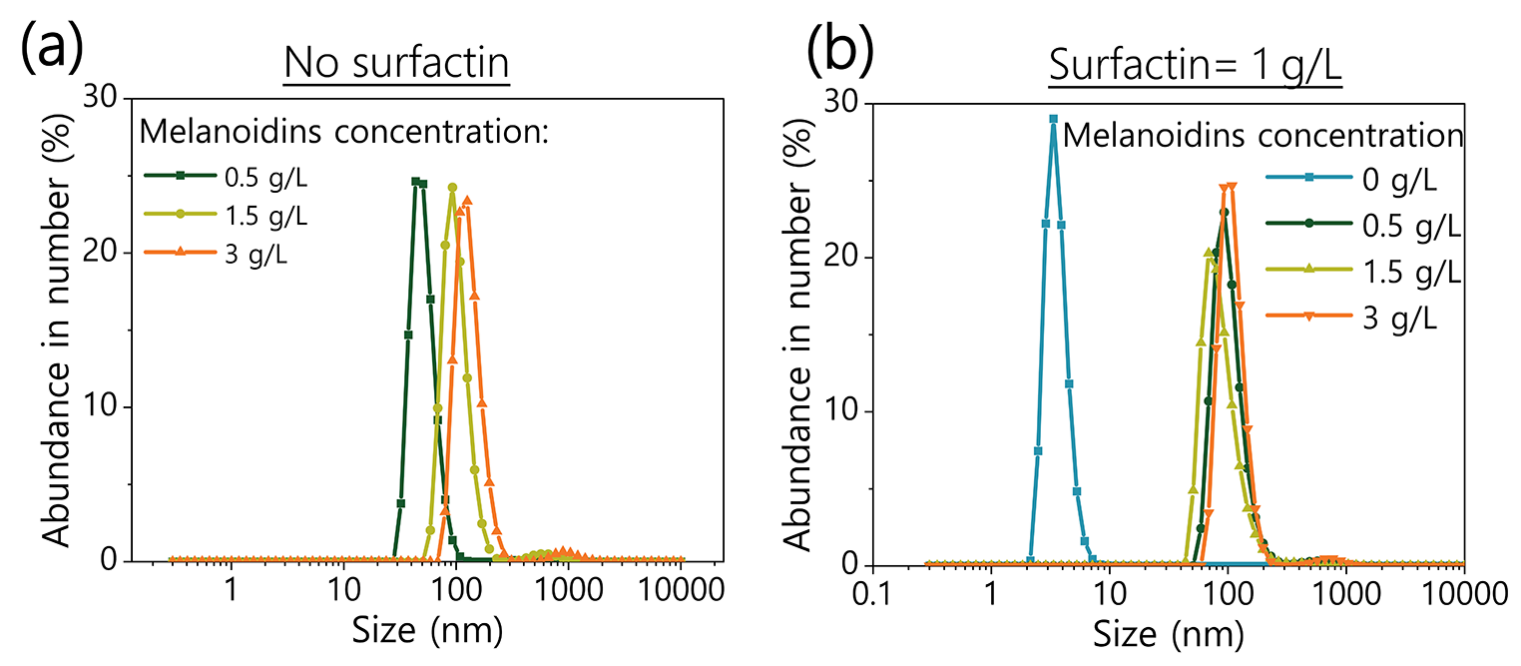
Size distribution (by their abundance in number) of the particles detected by dynamic light scattering in: **(a)** melanoidins solutions at concentrations ranging from 0.5 to 3 g/L, and **(b)** 1 g/L surfactin solutions containing between 0 and 3 g/L of melanoidins solutions

Next, we considered the possibility of an interaction between melanoidins aggregates and surfactin, since surfactants are molecules that tend to adsorb at interfaces. Melanoidins are macromolecules of variable size. In the particular case of coffee, high molecular weight melanoidins (> 12–14 kDa) are the more abundant fraction, representing a 59% of the total amount (Figure S4) (Wang et al. 2011b). In addition, melanoidins are known to self-aggregate forming particles of larger size (Pagán et al. 2012; Jiang et al. 2019). We used dynamic light scattering (DLS) to study the interaction between surfactin and melanoidins, testing the effect on the size of the detected micelles of surfactin and melanoidins aggregates. According to our results, the melanoidins extracted from SCG form aggregates between 50 and 200 nm (Fig. 5.a), sizes that are in agreement other works (Pagán et al. 2012; Lin et al. 2019). These sizes are only estimative though, given the poor fitting of the correlograms and the large fluctuation in the scattered light, which may indicate that these aggregates do not adopt a spherical form, that their configuration is dynamic or even some of them are precipitating, as previously reported (Pagán et al. 2012). Next, we analyzed the size of the micelles of surfactin at varying concentrations of melanoidins (Fig. 5.b). A monodisperse population of surfactin micelles of a size between 2 and 10 nm was detected when melanoidins were not added to the sample. However, when melanoidins were present, we observed the same particle distribution than the one found for the measurements without surfactin. In addition, no signal for the surfactin micelles in the range from 2 to 10 nm was detected in this case. This suggests that surfactin and melanoidins aggregates have a strong interaction, particularly taking into account that no free micelles of the biosurfactant were detected when melanoidins were present even at the high surfactin concentrations used for this experiment. This interaction can explain the high recovery rate of melanoidins with the acid precipitation used to purify surfactin, and more importantly, opens the possibility to develop of a co-purification process of both biomolecules that had not been previously reported.

#### Antioxidant capacity of CrS and CFS

SCG are rich in molecules with a potent antioxidant activity such as phenolic compounds or melanoidins. Accordingly, we have found that the SCG used in this work are antioxidant, in particular according to their DPPH activity (97.94 ± 1.89%), which is even higher than the activity found with the BHT solution used as a positive control (60.49 ± 1.89%). Next, we tested if the culture supernatant and crude surfactin extract retained this antioxidant power, by measuring their antioxidant activities with four different methods. Table 3 summarizes the concentration of the total phenolic compounds and the antioxidant capacities of CFS and CrS samples during the course of the culture. The evolution of the total phenol concentration resembled to that found for melanoidins, and the contribution of the SCG was clear if comparing these results with those for the blank experiments without SCG in the medium. In addition, the moderate increase in the concentration of phenols suggests that the microbial culture might improve their release from the SCG, as observed for melanoidins. Attending to the antioxidant capacity, CFS samples at 24 h showed higher antioxidant capacity compared to that of the culture media itself (t = 0 h) for all the four methods tested, in agreement with the concentration of phenols and melanoidins reported above. After these initial 24 h of culture, the DPPH activity and the inhibitor potential of the lipid peroxidation (ILP) decreased during the rest of the culture (albeit at different rates), while the Fe^2+^ chelating activity and iron reducing power (IRP) showed a slight increase.

**Table 3:** Total phenolic compounds (in mg/L) and antioxidant CFS and CrS.

	Total phenols (mg/L) ^a^	DPPH (%) ^a^	ILP (%) ^a^	Fe^2+^chelating activity (%) ^a^	IRP ^b^	
**CFS**						
24 h	508.69 ± 16.07	58.19 ± 2.14	88.30 ± 0.22	83.24 ± 0.91	2.58 ± 0.16	
48 h	602.97 ± 9.81	49.04 ± 2.45	87.69 ± 0.68	91.93 ± 4.32	2.80 ± 0.06	
72 h	531.94 ± 15.70	7.63 ± 0.59	80.80 ± 1.81	88.85 ± 6.05	2.97 ± 0.18	
96 h	549.37 ± 16.30	18.80 ± 0.91	78.20 ± 2.66	87.47 ± 9.56	3.33 ± 0.07	
120 h	533.71 ± 11.80	17.47 ± 3.86	76.95 ± 1.06	95.65 ± 0.06	3.47 ± 0.10	
Blank 48 h	195.22 ± 0.76	31.34 ± 2.48	60.68 ± 6.63	92.39 ± 3.27	1.50 ± 0.12	
Blank 0 h	156.26 ± 13.61	53.08 ± 4.18	76.93 ± 6.99	29.07 ± 7.47	0.90 ± 0.10	
**CrS**						
24 h	21.36 ± 3.55	80.40 ± 0.79	67.47 ± 0.94	40.76 ± 4.20	0.14 ± 0.05	
48 h	25.57 ± 6.64	80.79 ± 1.44	69.53 ± 0.65	37.14 ± 11.08	0.33 ± 0.04	
72 h	-	60.94 ± 3.04	54.64 ± 6.28	38.34 ± 5.05	0.04 ± 0.06	
96 h	15.66 ± 1.19	85.00 ± 0.95	75.02 ± 0.86	59.38 ± 0.21	0.02 ± 0.02	
120 h	5.61 ± 0.28	64.19 ± 1.27	70.23 ± 0.62	70.25 ± 7.46	0.04 ± 0.05	
Blank 48 h	25.37 ± 0.93	28.52 ± 0.96	58.24 ± 2.59	18.89 ± 1.60	0.18 ± 0.07	
**Spent coffe grounds**						
	0.18 ± 0.02	97.94 ± 0.10	84.23 ± 0.63	39.42 ± 1.03	144.6 ± 9.3	
**Positive control** ^c^						
	---	60.49 ± 1.89	90.55 ± 1.05	77.26 ± 4.85	---	

a. Results for CFS are referred to one mL of sample, and for the solid samples (CrS, SCG) are expressed per mL of solutions of the solid analyzed at a concentration of 1 mg/mL

b. Results for CFS are referred to one mL of sample and for solid samples (CrS, SCG) are expressed per g of sample

c. Positive controls. For DPPH: BHT 1 mg/mL; for ILP: BHT 1 mg/mL; for Fe^2+^ chelating power: EDTA 100 µg/mL

On the other hand, the concentration of total phenolic compounds in the CrS remained constant during the first 48 h of culture, and showed a decreasing trend after that time. Furthermore, in this case, there was not a significant difference in the amount of phenols recovered compared to the blank experiment without SCG. This indicates that, despite being present at high concentration in the culture supernatant, the acid precipitation used to recover the surfactin from the CFS is not an efficient approach to recover the phenolic compounds. Nevertheless, other methods such as tangential flow filtration could be a suitable option to co-purify both, melanoidins and phenols (Rufián-Henares and de La Cueva 2009; Pastoriza and Rufián-Henares 2014).

Interestingly, despite the reduced concentration of phenols in the CrS, their antioxidant capacity was considerably higher than that of the control. Again, the antioxidant capacity remained almost constant during the first 48 h of culture according to the four methods tested. After that time, the Fe^2+^ chelating capacity of CrS showed an increasing trend. In contrast, a reduction of 15% in the DPPH activity was detected between samples taken at 24 and 120 h of culture, while no significant differences were found in the ILP antioxidant capacity values over time. Finally, the antioxidant capacity values of CrS obtained by the IRP method were low, ranging from 0.14 to 0.04 mg gallic acid/g CrS for 24 and 120 h of culture respectively.

## Discussion

In this work we show that spent coffee grounds (SCG) can be valorized by using them as the carbon source in cultures of *B. subtilis*. The immediate benefit of that was the production of the high added value biosurfactant surfactin, which reached concentrations up to 8.8 mg/L in the supernatant after 96 h of culture. Despite being low, this surfactin yield is higher than previous results of our group using other agro-industrial wastes such as olive oil mill waste or frying oils as carbon sources (Moya Ramírez et al. 2015; Maass et al. 2016; Valenzuela Ávila et al. 2019). Therefore, SCG seems to be a good candidate as raw material for the production of surfactin, particularly if considering that the yields reported here could be considerably improved by a pretreatment of the waste to increase the bioavailability of the polymeric carbohydrates (Moya Ramírez et al. 2016; Pérez-Burillo et al. 2019). It is also important to note that almost 50% of the soluble carbohydrates in the SCG medium remained unconsumed. This represents an opportunity for further improvement of the process by developing strains with a wider spectrum of metabolizable sugars (Dvořák and de Lorenzo 2018).

The crude surfactin obtained showed good interfacial and emulsifying properties. Besides, we found that the presence of SCG in the medium exerted an effect on the surfactin congener distribution. Interestingly, we report for first time that a microbial culture actively contributes to the recovery of melanoidins from SCG, and that these can be co-extracted with surfactin to a high extent. This could be due to the interaction between the biosurfactant and melanoidins, as we have observed by DLS. Moreover, we have analyzed in depth the concentration of melanoidins and the antioxidant properties of the cell-free supernatant and crude surfactin extract. The correlation between the concentration of phenols and DPPH, the inhibition of lipid peroxidation and chelating activity tests was poor, implying that molecules on CrS other than phenols could be responsible of part of its antioxidant capacity. Among them, melanoidins are likely contributing to that since, as mentioned above, they are known to have antioxidant properties. In addition, the surfactin itself could also be partially responsible since it was found to have antioxidant power (Kiran et al. 2017). It is important to consider that the microbial growth substantially modifies the composition of the culture media, and consequently, the variety and concentration of the components in the CFS and CrS that can have an influence on their antioxidant activities. Our results suggest that the concentration of phenolic compounds, melanoidins and biosurfactant contribute to the antioxidant activities in a complex manner. We can conclude nonetheless that: (i) in a general way, the culture time affected negatively the antioxidant capacity values determined by DPPH; (ii) no important changes in the antioxidant activity were detected with ILP method in the samples taken at different times.

Altogether, we consider that these results can pave the way for an effective and novel valorization route of SCG. This route would combine the production of high added value biomolecules such as surfactin from the carbon fraction accessible with the improved recovery of other valuable molecules already present in SCG such as melanoidins or phenolic compounds. Indeed, melanoidins represent an important potential revenue and as such there is a growing industrial interest in their commercialization (Iriondo-DeHond et al. 2021). More specifically, we estimate that pure melanoidin sales revenue could reach 600 $/Kg, with production costs being around 23 $/ (Peters et al. 2014; Manuel and Agudo 2017). However, melanoidins isolated from SCG are not yet commercially available as food additive or for their use as ingredient in other formulations. Therefore, more legislations and deeper studies on the effects of their consumption would be needed. On the other hand, surfactin (and biosurfactants in general), have already a well-stablished and expanding market share, which is expected to reach 2.60 billion USD by 2027 (Global Market Insights Inc 2020; Pardhi et al. 2022). In particular surfactin of a purity greater than 90%, can be purchased from 300 $/Kg.

In addition to being high added-value substances both, melanoidins and surfactin, have high efficiencies for some applications, and therefore are active at lower concentrations compared to their synthetic counterparts. For example, coffee melanoidins have a strong antioxidant capacity, 1000-times higher than that of vitamin C or chlorogenic acid, commonly used as antioxidant additives (Delgado-Andrade et al. 2005). They also have antihypertensive activity and, although such bioactivity is 500-times lower than a standard antihypertensive drug such as captopril, the usual dose in a food serving (around 100–500 mg) have the same antihypertensive activity than a dose of captopril (Rufián-Henares and Morales 2007; Iriondo-DeHond et al. 2021). Likewise, surfactin has a strong antimicrobial activity and is a powerful biological preservative, while showing a low toxicity in mice (Heerklotz and Seelig 2001; Chen et al. 2022). It has also a very low CMC value, two orders of magnitude lower than the CMC of the majority of synthetic surfactants (Santos et al. 2019). This means that it is highly efficient, and thus ideal for environmental applications such as oil spill remediation, since lower doses of surfactin will be required compared to other surfactants with higher CMCs (Patel et al. 2018). Consequently, the final product obtained in this work combining both biomolecules could find applications of interests ranging from those of the alimentary industry to the protection of the environment.

## Electronic supplementary material

Below is the link to the electronic supplementary material.

Supplementary Material 1: Figures S1 to S5 show the UPLC-MS chromatograms of the culture supernatant, the calibration curves used to quantify surfactin and melanoidins, a gel permeation chromatogram of melanoidins extracted from SCG, and the CMC curves of commercial surfactin in the presence and absence of melanoidins

## Data Availability

Datasets generated during this study are available on request from the corresponding author.
