# Supplementary material for: Spent coffee grounds as feedstock for the production of biosurfactants and the improved recovery of melanoidins
Source: World J Microbiol Biotechnol. 2023 Jul 18;39(9):254. doi: 10.1007/s11274-023-03698-x (PMC10353961; doi:10.1007/s11274-023-03698-x)
Supplement: Supplementary file 1 — Supplementary Material 1: Figures S1 to S5 show the UPLC-MS chromatograms of the culture supernatant, the calibration curves used to quantify surfactin and melanoidins, a gel permeation chromatogram of melanoidins extracted from SCG, and the CMC curves of commercial surfactin in the presence and absence of melanoidins [file 11274_2023_3698_MOESM1_ESM.docx]

**Supplementary information for:**

Spent coffee grounds as feedstock for the production of biosurfactants and the improved recovery of melanoidins

Ignacio Moya-Ramirez^a^*, María Encarnación Pegalajar Robles, ^a^ Michele Debiasi Alberton, ^b^ José Á. Rufián-Henares, ^c^ Alejandro Fernández-Arteaga, ^a^ Miguel Garcia-Roman, ^a^ Deisi Altmajer Vaz. ^a^

1. Departmento de Ingeniería Química, Universidad de Granada, 18071 Avda. Fuentenueva s/n, Granada, Spain.
2. Departamento de Ciências Farmacêuticas, Universidade Regional de Blumenau, Brasil.
3. Departamento de Nutrición y Bromatología, Instituto de Nutrición y Tecnología de los Alimentos, Centro de Investigación Biomédica and Instituto de Investigación Biosanitaria ibs.GRANADA, Universidad de Granada, 18100 Granada, Spain.

* Correspondence to Ignacio Moya Ramírez: ignaciomr@ugr.es

6 pages

5 figures


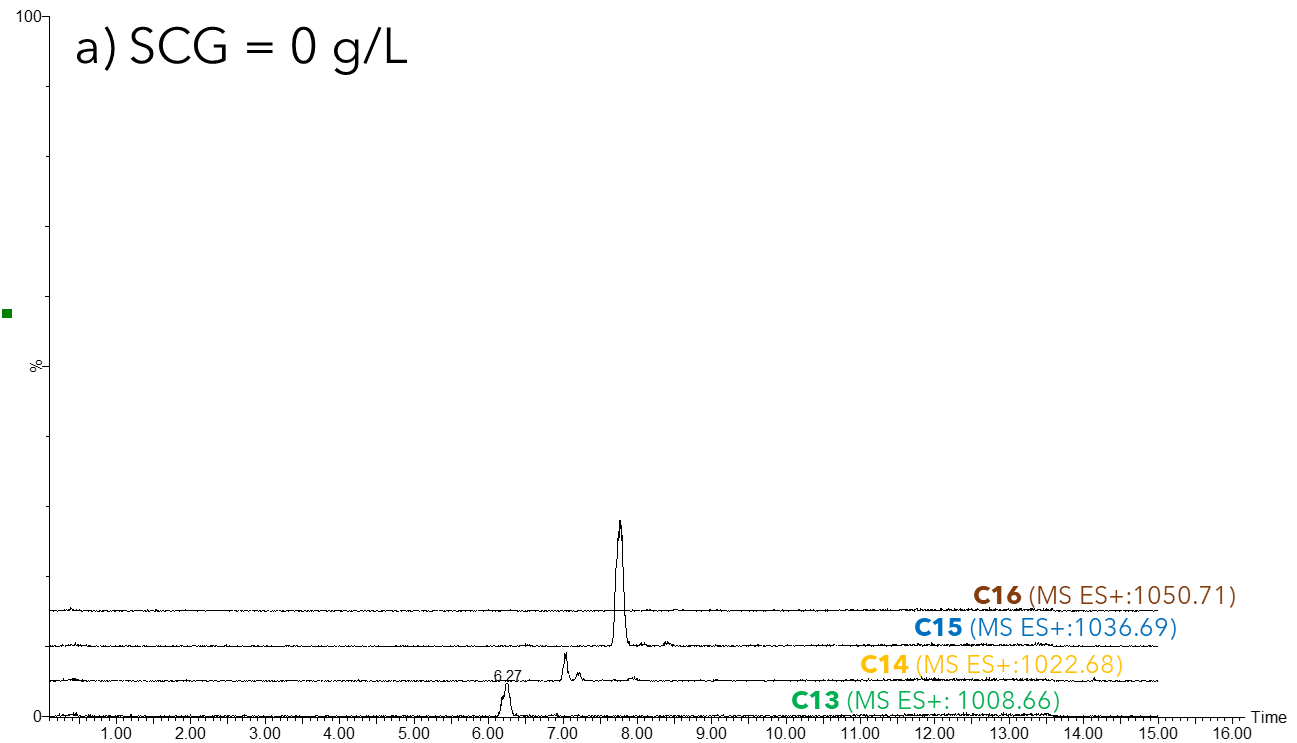


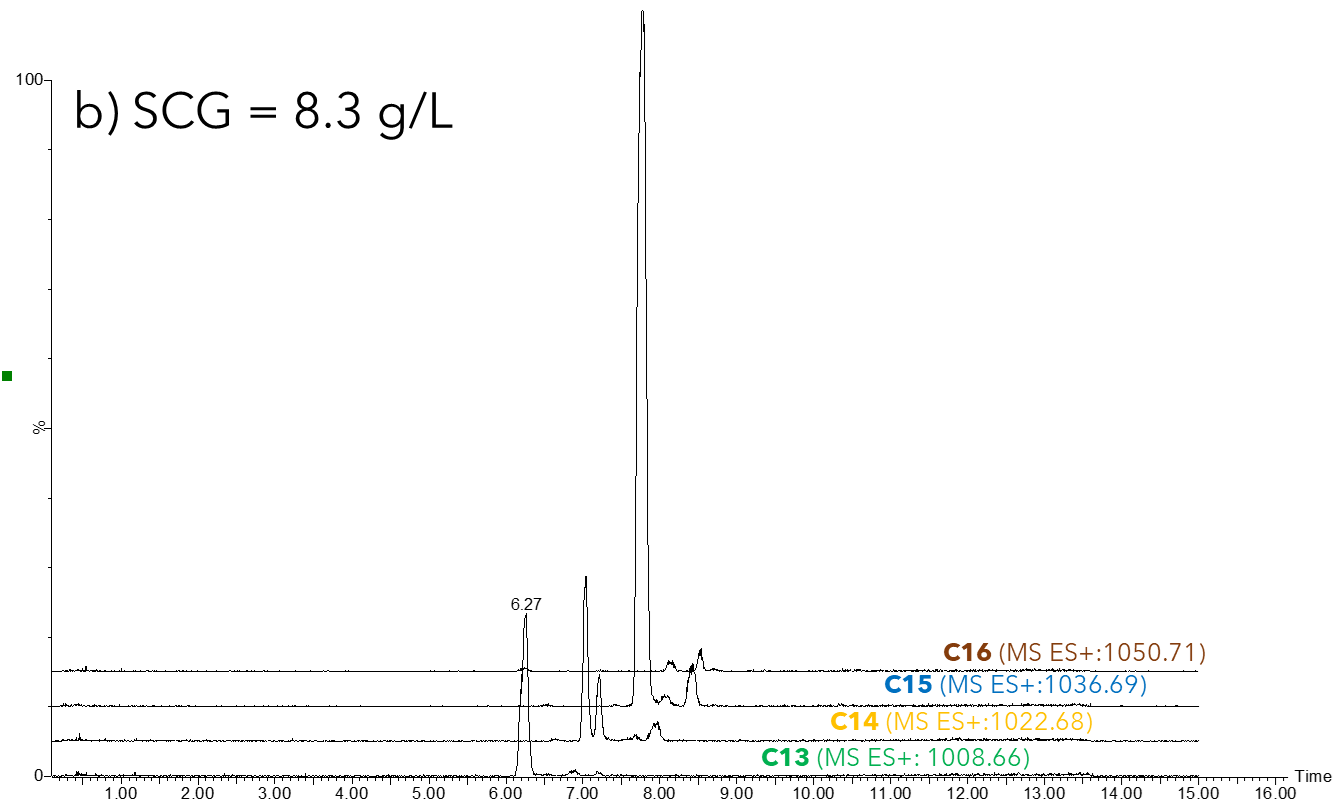


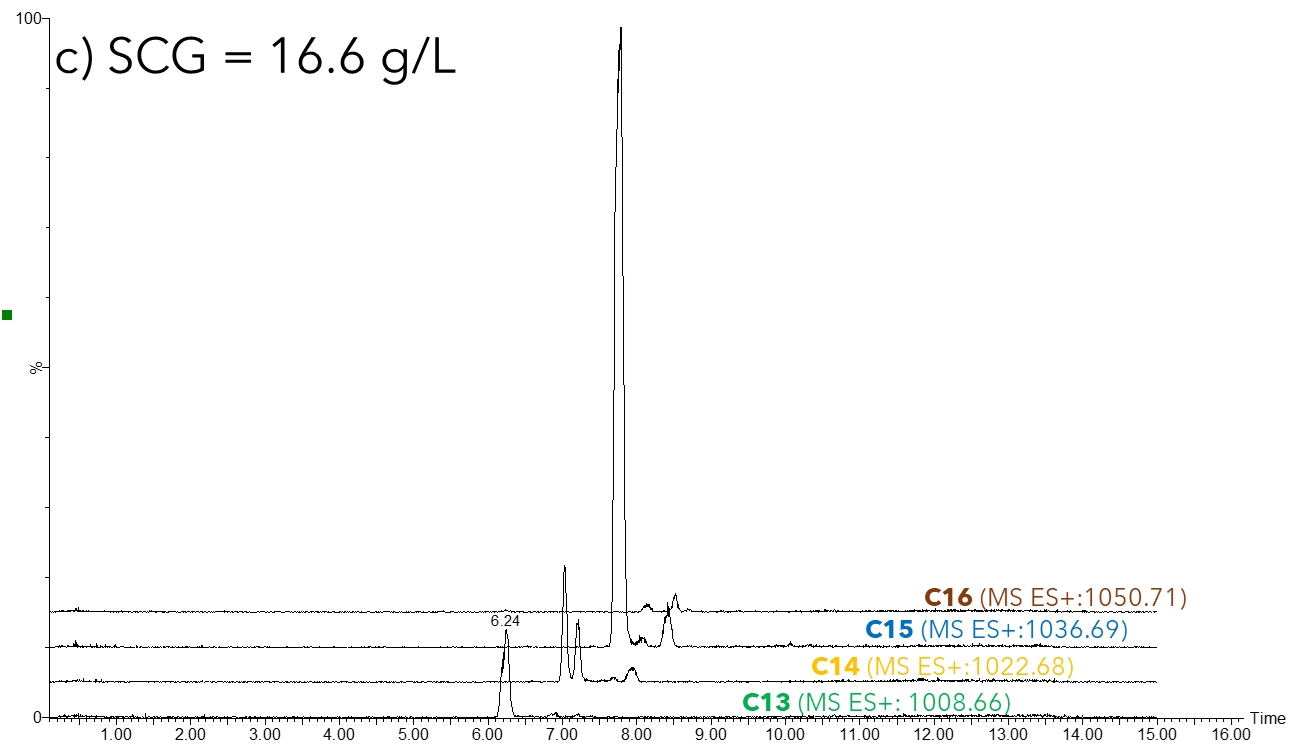


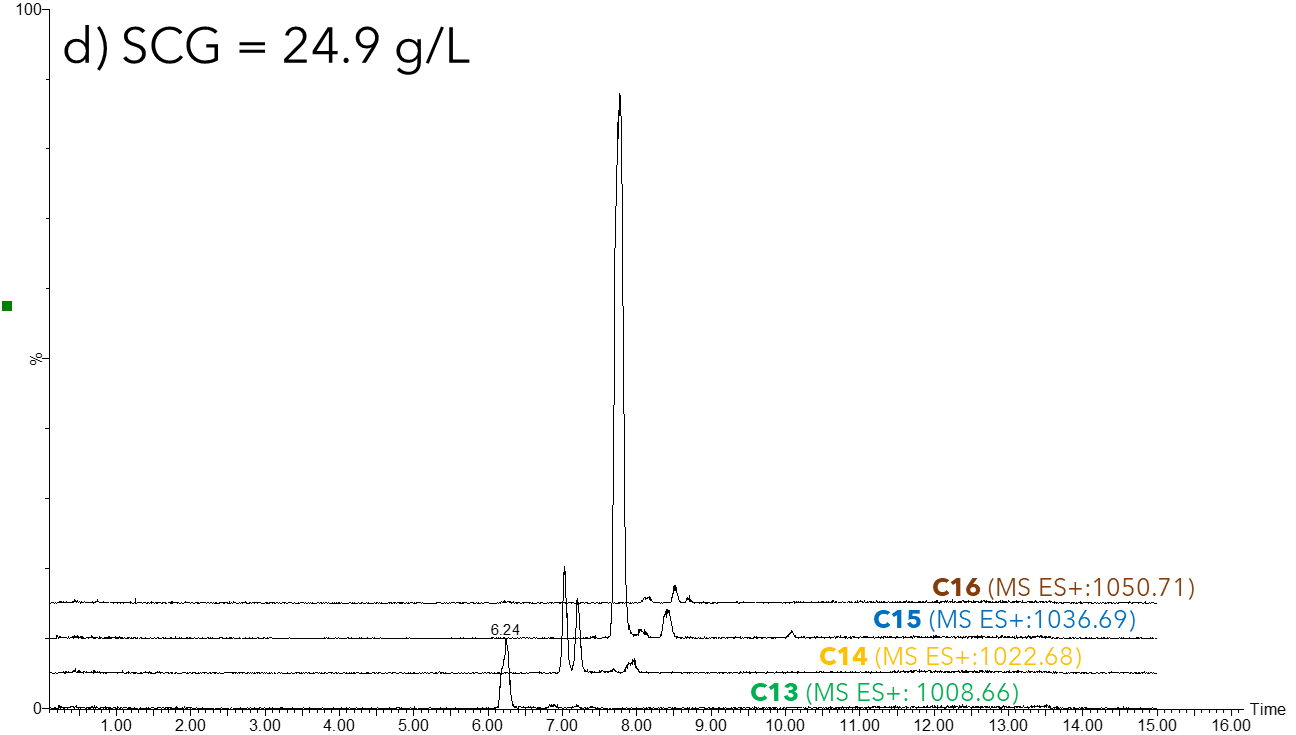


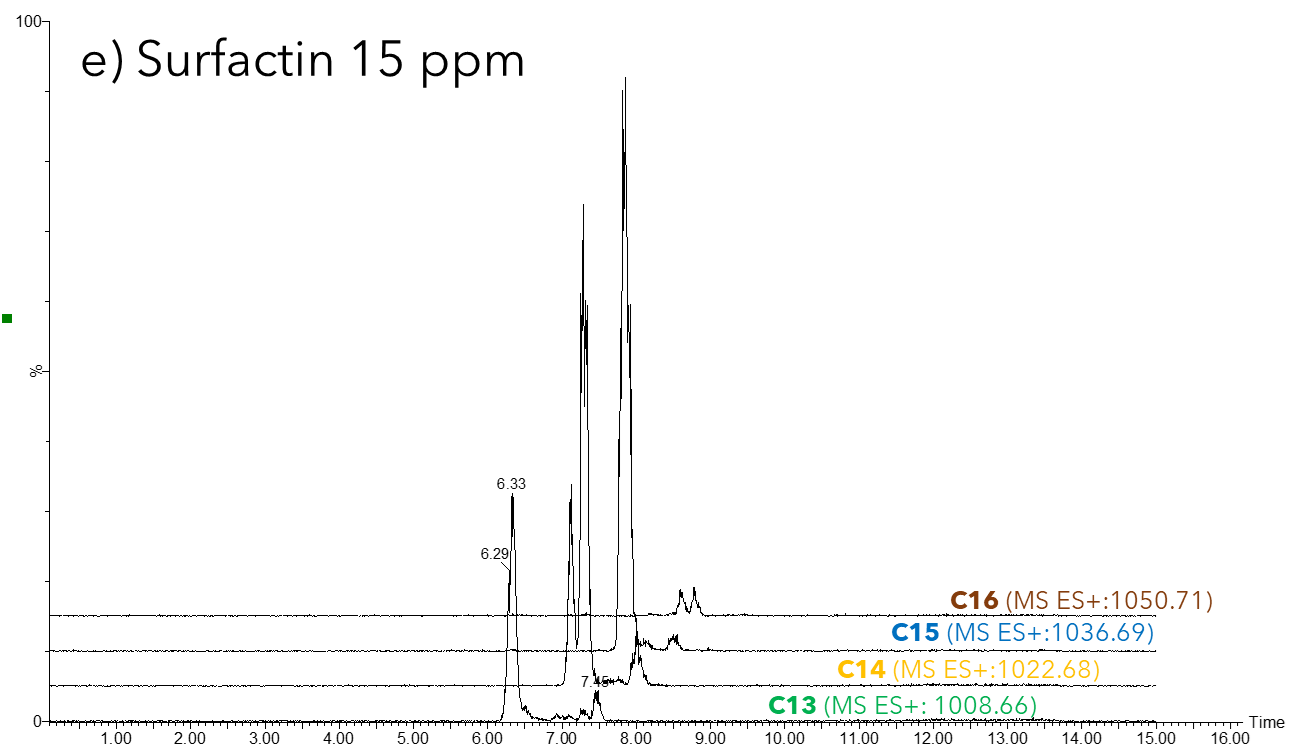


**Figure S.1.** UPLC-MS chromatograms of the culture supernatant after 96 h of culture for different spent coffee grounds (SCG) concentrations: a) 0 g/L, b) 8.3 g/L, c) 16.6 g/L, d) 24.9 g/L; together with a surfactin standard at 15 ppm (e). Each figure shows the MS ES + chromatograms of the four surfactin congeners detected (C13 to C16), obtained by extracting the MS signal at their molecular weights (indicated inside the parentheses).


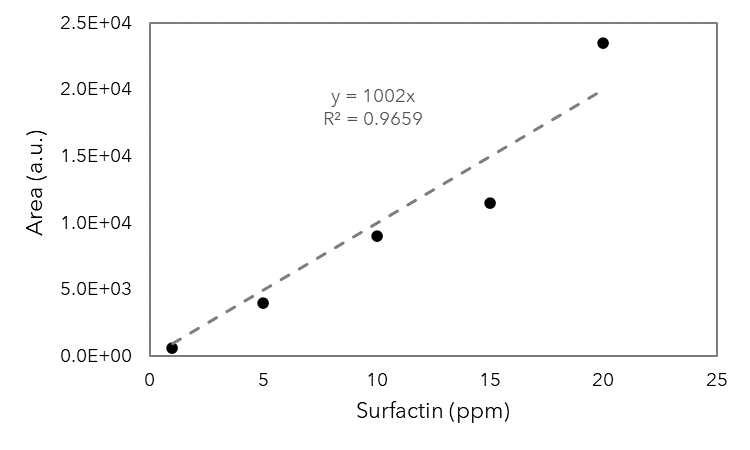


**Figure S.2.** UPLC-MS surfactin concentration calibration curve prepared with solutions of HPLC-grade surfactin at different concentrations. The areas represent the sum of the areas of the four surfactin congeners detected.


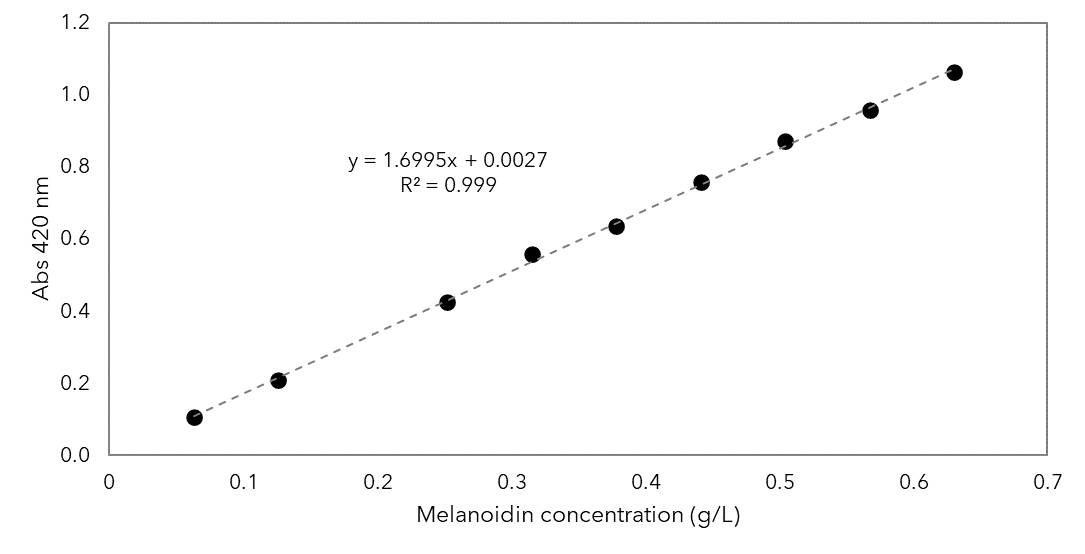


**Figure S.3**. Calibration curve prepared with extracted melanoidins from SCG as previously described by Rufián-Henares and de la Cueva,^1^ by measurement of the absorbance of the samples at at 420 nm.


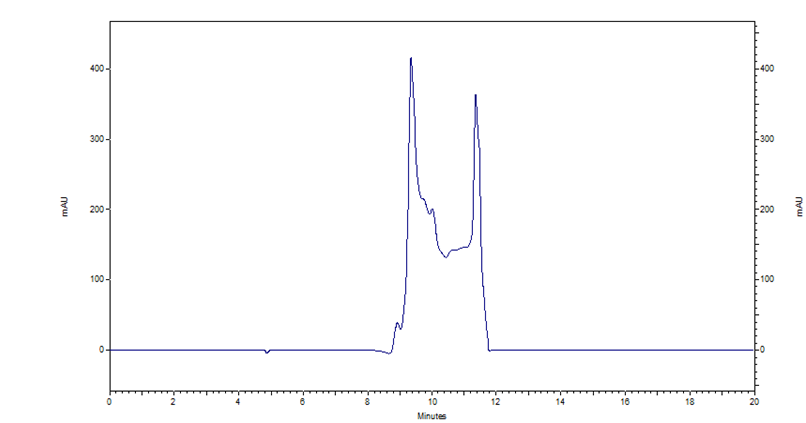


**Figure S.4.** Gel permeation chromatogram of melanoidins extracted from SCG, according to the process described by Morales (2002).^2^ Briefly, 100 mg of SCG melanoidins were dissolved and vortexed for 2 minutes with 1 mL of 50 mM sodium phosphate (pH = 7). The solution was then centrifuged at 13300 rpm for 5 minutes and filtered through a 0.22 mm filter. The samples were analyzed in an Accela 600 HPLC (Thermo Fisher Scientific, Waltham, MA, USA) equipped with a quaternary pump, column oven, autosampler, a PDA detector and a Discovery Bio GFC 150 column (30 cm x 4.6 mm, 5 mm, 150Å) from Supelco (Bellefonte, MA, USA). Samples (20 mL) were injected and eluted in a mobile phase of distilled water at a flow rate of 0.8 ml/min. Reads were taken at 420 nm. According to their retention times, SCG melanoidins have a molecular weight ranging between 21 and 28 kDa.

**Figure S. 5**. The surface tension of succesive dilutions of comercial surfactin (Kaneka, purity >90 %), ploted as the log_10_ of its concentraion in mg/L. The effect of the presence of melanoidins on the CMC of surfactin was analysed. For that, surfactin samples were diluted in 0.1 M phosphate buffer at pH 7.4, either in the absence of melanodinis or with 2.75 g/L of melanoidins. As can be observed, for both cases, the CMC is around 10 mg/L and a ST value of 28 mN/m.

**Additional references**

(1) Rufián-Henares, J. A.; de La Cueva, S. P. Antimicrobial Activity of Coffee Melanoidins—A Study of Their Metal-Chelating Properties. *Journal of Agricultural and Food Chemistry* **2009**, *57* (2), 432–438.

(2) Morales, F. J. Application of Capillary Zone Electrophoresis to the Study of Food and Food-Model Melanoidins. *Food Chemistry* **2002**, *76* (3), 363–369.
